# Dissimilar Phonemes Create a Contextual Interference Effect During a Nonword Repetition Task

**DOI:** 10.3389/fpsyg.2020.585745

**Published:** 2020-11-10

**Authors:** Kimberly M. Meigh, Elisabeth Kee

**Affiliations:** Speech Motor Control Lab, Department of Communication Sciences and Disorders, West Virginia University, Morgantown, WV, United States

**Keywords:** speech, motor learning, contextual interference, phoneme, phonemic complexity

## Abstract

**Purpose**: The contextual interference effect is a motor learning phenomenon where conditions that decrease overall learning during practice enhance overall learning with new tasks. In the limb literature, this effect is observed when different practice conditions are used (e.g., blocked vs. random practice schedules). In speech motor learning, contextual interference effects are mixed. The differences observed during speech motor learning may be due to the stimuli used. We hypothesized that dissimilar phonemes might create interference during speech motor learning, such that training accuracy would decrease. However, generalization accuracy would increase compared to practice with nonwords containing similar phonemes.

**Method**: Thirty young adults with typical speech and hearing participated in a motor learning study using a cross-over design. Participants engaged in nonword repetition training followed by an immediate retention and transfer task with two sets of nonwords: nonwords with similar phonemes and nonwords with dissimilar phonemes. Percent consonants correct were calculated to examine the effects of the two different types of nonwords based on the stage of skill acquisition.

**Results**: A contextual interference effect was observed in this study using nonwords that varied in phonemic similarity. Nonwords with similar phonemes were produced with greater accuracy during the training stage of skill acquisition, and nonwords with dissimilar phonemes were produced with greater accuracy during the transfer stage.

**Conclusion**: The proposed hypothesis for this study was met – practicing nonwords with dissimilar phonemes resulted in greater accuracy in the transfer phase of this experiment. Results indicate that phonemic dissimilarity produced contextual interference and influenced speech motor learning. These results indicate that the linguistic properties of stimuli must be factored into speech motor learning. Future research should explore if other linguistic variables interact with variables of motor learning to enhance speech practice and generalization outcomes.

## Introduction

The contextual interference effect is a paradoxical phenomenon. Conditions that decrease learning during practice (interference variables) increase overall learning when attempting new tasks (Magill and Hall, 1990; Lee et al., 1992). The most well-researched interference condition investigated in the limb motor learning literature is practice schedule (i.e., blocked vs. random practice). During blocked practice, one motor skill is repetitively practiced before moving onto a second motor skill. In contrast, during random practice, both motor skills are practiced. The order of practice is variable between the two skills. Random practice, when compared to blocked practice, results in better generalization, or transfer, to new movements (Shea and Morgan, 1979; Magill and Hall, 1990). Evaluation of practice schedules during speech motor learning has yielded mixed results (Scheiner et al., 2014; Jones and Croot, 2016). One reason for this may be that speech is a unique motor act influenced by motor and linguistic processing. Thus, the properties of the stimuli used during training may influence speech motor learning.

Empirical evidence of practice schedules creating interference effects in typical speakers during speech motor learning is mixed. Adams and Page (2000) investigated the effect of feedback and practice schedule during repetitions of the phrase “Buy Bobby a puppy.” Practice schedules were varied by temporal duration, with results revealing no difference in absolute error between blocked and random practice schedules at the end of the training. However, lower absolute error rates were reported for the random practice group during a retention task. Wong et al. (2013) conducted a similar experiment in Cantonese to evaluate blocked, random, and mixed practice schedules (e.g., blocked-then-random schedule). No significant differences in utterance duration were observed between the random- and blocked-only practice schedule groups at the end of training or during the retention tasks. Transfer performance was evaluated using a dual-task paradigm and revealed the secondary task less adversely influenced subjects who trained using a mixed practice schedule. Jones and Croot evaluated mixed and singular practice schedules using tongue twisters, e.g., “Bell Pod Pun Boot”(Jones and Croot, 2016, p. 358). They reported no significant differences in accuracy between blocked and random practice schedules by participants following training or retention tests. However, blocked-random and random practice schedules generally facilitated better retention of learning. Additionally, these authors reported differences in errors based on the specific tongue twister used during blocked practice in the training and retention conditions.

Practice schedules have also been evaluated using nonword stimuli. Scheiner et al. (2014) constructed four nonwords that varied by the number of syllables and phonemes. Following training, the blocked practice group produced these nonwords with significantly higher accuracy, shorter duration, and lower variability compared to the random practice group. The random practice group was significantly more accurate in producing the nonwords during the retention task. However, no significant difference in nonword duration or variability between the practice groups was reported. *Post hoc* item analyses following training and retention revealed specific nonwords were produced more accurately, quickly, and with less overall variation during each phase of the experiment compared to the other trained nonwords. Kaipa et al. (2017) used nonword phrases to evaluate practice variability and practice schedule in younger and older adults. Only the retention phase of motor learning was evaluated with results indicating significantly better spatial accuracy (as measured by percent phonemes correct, PPC) for older subjects using the random practice schedule. No significant difference in PPC scores was reported for younger subjects regardless of practice schedule. Temporal accuracy, as measured by mean phi correlation, revealed younger participants were able to learn the nonword phrases significantly better than older adults regardless of practice schedule.

The varying degrees of contextual interference reported in the speech motor learning literature may stem from the different interpretations regarding the fundamental programming unit, i.e., speech motor programs, studied during speech motor learning. Currently, there is no consensus on the size of the programming unit for speech production. Linguistic and speech production models have postulated syllables as a programming unit (e.g., Levelt et al., 1999; Cholin et al., 2006). However, there is also significant evidence to suggest smaller programming units, e.g., phonemes, may influence speech production more overtly (e.g., Meigh, 2017; Liu et al., 2018; Meigh et al., 2018). These different interpretations influence the construction of stimuli and the variables controlled across stimuli sets in experimental studies of speech motor learning. Of the reviewed studies, two explicitly noted the influence of specific phoneme factors on the outcomes of their study. Scheiner et al. (2014, p. 35) observed certain levels of complexity in their stimuli were not controlled, e.g., phoneme markedness. Similarly, Jones and Croot (2016) controlled the initial phoneme pairs in their tongue twisters for sequence and articulation positions. They also reported an imbalance in their stimuli based on place of articulation, which may have influenced the difficulty of the stimuli.

These potential influences suggest that selecting specific phonemes may alter the amount of interference present during learning. This effect is reported in the perceptual learning literature involving second language acquisition using high variability pronunciation training (HVPT). During HVPT, minimal pairs containing nonnative phonemes are trained until speakers can discriminate between similar sounds (for a review, see Barriuso and Hayes-Harb, 2018; Thomson, 2018). Nishi and Kewley-Port (2007) reported better generalization to new English vowels following HVPT training with a full set of vowels than a subset of three vowels in native Japanese speakers. These generalization results were in contrast to observing better acquisition during training with the smaller subset of vowels. A follow-up study a year later replicated these findings in Korean speakers indicating that training with larger stimuli sets provided greater overall generalization and learning compared to smaller sets (Nishi and Kewley-Port, 2008). By increasing the variability of conditions, such as the number of contrasting phonemes, Nishi and Kewley-Port (2007, 2008) observed a contextual interference effect in their perceptual learning studies.

This study aimed to investigate whether varying the number of phoneme contrasts would produce a contextual interference effect when practice schedules were held constant during training. Using a cross-over design, participants engaged in two speech motor learning tasks with different nonword stimuli that varied in phoneme similarity. In this study, phoneme similarity was defined in two ways: (1) by the number of repeated phonemes within a nonword, and (2) by psycholinguistic variables evaluated in previous studies (e.g., biphone probability, articulatory features). Following both motor learning tasks, we evaluated participants’ accuracy based on the type of stimuli (similar and dissimilar) and motor learning phase (training and generalization). Our first hypothesis was that participants would demonstrate learning of similar and dissimilar nonword stimuli as the result of practice during the training phase of the experiment. Our second hypothesis, presented in two parts, predicted the contextual interference effect. Participants practicing similar nonwords during training would have greater accuracy at the end of training than when practicing dissimilar nonwords, with the reverse pattern observed during generalization.

### Participants

Thirty adults (23 female, seven male) between 18 and 31 years of age (*M* = 21, *SD* = 3) participated in this study. All participants were native speakers of English, and the primary investigators screened their speech and hearing skills. Participants’ conversational speech was evaluated for articulation and fluency errors. The Test of Minimal Articulation sentence and reading screening subtests (Secord, 1981), as well as an oral motor examination, were used to rule out speech disorders. Hearing acuity was screened using pure tone thresholds at 35 dB HL at 500, 1,000, 2,000, and 4,000 Hz in at least one ear (American Speech-Language-Hearing Association, 1990). Speech discrimination abilities were screened using the Northwestern University Auditory Test No. 6 word list (Tillman and Carhart, 1966). All participants were required to identify 98% of all words correctly. Working memory capacity and phonological processing abilities were documented using the Digit Span and Nonword Repetition Subtests from the Comprehensive Test of Phonological Processing, Second Edition (CTOPP; Wagner et al., 2013). These working memory measures were not used to rule out participants but to classify the participants’ overall memory processing capabilities: Digit Span percentile (*M* = 50.39, *SD* = 19.68), Nonword Repetition percentile (*M* = 42.09, *SD* = 23.42).

All participants signed informed consent documents approved by the West Virginia Institutional Review Board (IRB) before initiating the screening procedures outlined above. Participants who were eligible for the study based on the above screening procedures were compensated for their participation in this study. All procedures outlined (screening and experimental) were approved by the West Virginia University (WVU) IRB and are in accordance with all guidelines and regulations related to behavioral experiments with human subjects.

### Stimuli

The stimuli used in this study consisted of 40 nonwords divided into two sets of 20 based on phoneme repetition (Kendall et al., 2005; Meigh, 2017). The set of nonwords that had the most phoneme repetitions was considered the “similar” set. The other set was considered the “dissimilar” set. Each set of 20 nonwords was then randomly split into two sets of 10 nonwords to create a “training” and “transfer” set. All nonwords consisted of three syllables (CV|CV|CVC) comprised of novel combinations of phonemes that followed English phonotactic rules. Syllable stress for all nonwords was on the first or second syllable. Table 1 details the full list of stimuli.

**Table 1:** Stimuli categorized by type (similar and dissimilar) and motor learning phase (training and transfer).

SIMILAR		DISSIMILAR	
Training	Transfer	Training	Transfer
/teI**næ**rok/	/zæ**ʃɔ**ʤəθ/	**/ʃɔ**ʤəzɔd/	**/næ**θodæp/
/**kæ**θotæs/	/ʤə**zɔ**zæk/	**/vu**zæʃɔm/	**/dɔ**ʤəzɔd/
/**sæ**θodæk/	/zæ**nɔ**ʤəθ/	**/fo**zæʃɔd/	/sʌ**ve**Inæθ/
/**zo**teInav/	/ʤʌ**nɔ**zæk/	**/ko**zæʃɔm/	/na**sæ**θoʃ/
/za**ʃɔ**ʤəz/	/**θʌ**rasæθ/	/ra**sæ**θon/	/vi**ʃə**dæk/
**/næ**teIrok/	/**ʃɔ**zæʤəθ/	/gi**bɪ**ðɪb/	/**bɪ**ðeItʃug/
/θo**kæ**tæs/	/**zɔ**ʤəzæk/	/ʒi**bʊ**tʃeIð/	/**gi**gʊðib/
/θo**sæ**dæk/	/**nɔ**zæʤəθ/	/**tʃe**Iðugʊʒ/	/**tʃe**Ijiwɪʒ/
/teI**zo**nav/	/**nɔ**ʤʌzæk/	/**ʒʊ**gijub/	/bʊ**tʃi**tʃeIʒ/
/**ʃɔ**zaʤəz/	/ra**θʌ**sæθ/	/gʊ**gi**ðʊtʃ/	/tʃʊ**tʃu**bɪʒ/

All stressed syllables are bolded.

Several parameters were used to distinguish similar and dissimilar sets. The previous review of the literature suggested that multiple phonemic factors may influence speech motor learning (Table 2). The average number of different phonemes within a set was calculated based on a single occurrence of a phoneme within a nonword (i.e., repetitions of phonemes were not included). Average phonotactic probabilities were calculated using the University of Kansas’ phonotactic probability online calculator (Vitevitch and Luce, 2004). Position-specific probabilities relate to the frequency of a given phoneme to appear in a specific position in all words of the English language. In contrast, biphone probabilities describe the probability of two adjacent phonemes occurring within a word together (Vitevitch and Luce, 2004). Intraword similarity values were calculated by counting the number of shared consonants or vowels within a nonword and then averaging all values within stimuli sets. A feature-based analysis was also conducted for each set of stimuli based on consonant voice, place and manner, and vowel height and advancement to provide further evidence of similarity/dissimilarity between sets (Rogers and Storkel, 1998; Bailey and Hahn, 2005). These percentages included all repetitions of a phoneme within a word.

**Table 2:** Stimuli set characteristics.

	SIMILAR		DISSIMILAR	
	Training	Transfer	Training	Transfer
Average number of different phonemes	19	13	28	28
Average phonotatic probabilities: position-Specific	1.25	1.21	1.17	1.18
Average phonotatic probabilities: biphone	1.11	1.01	1	1
Intraword similarity: consonants	4	8	0	8
Intraword similarity: vowels	8	0	4	4
**Percentage of consonant features**				
Voiced	55	40	28	28
Voiceless	43	60	73	73
Place: bilabial	0	0	30	20
Place: labiodental	20	0	20	10
Place: interdental	20	27	20	17
Place: alveolar	55	53	17	25
Place: palatal	20	40	27	33
Place: velar	20	40	33	13
Manner: stop	35	40	30	33
Manner: fricative	60	44	50	40
Manner: affricate	20	27	10	20
Manner: liquid	20	10	10	0
Manner: glide	0	0	10	10
Manner: nasal	13	20	30	15
**Percentage of vowel features**				
High	0	0	43	43
Mid	53	47	25	33
Low	47	53	25	23
Front	43	33	40	57
Central	20	33	10	15
Back	50	50	53	33

### Study Design

**Figure 1:**
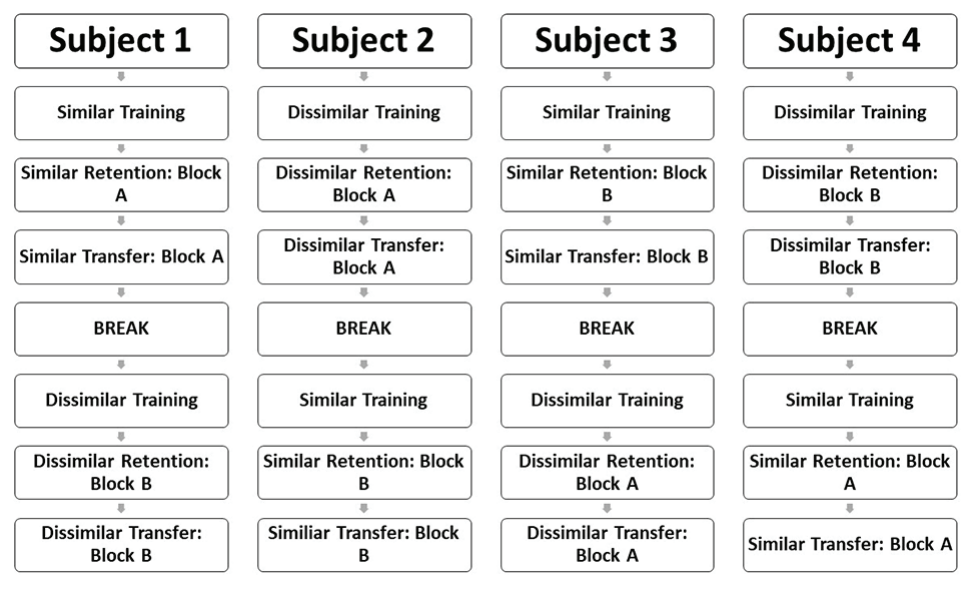
Schematic depiction of the nonword repetition task across participants using a cross-over design.

A randomized, cross-over design was used to evaluate the effects of phoneme similarity on speech motor learning. Participants were randomly assigned to start training with either similar or dissimilar nonwords. Once the training and generalization phases were complete with the first set of nonwords, a second nonword repetition task with the other nonwords was initiated following a 5–10-min break. As noted previously, nonwords were randomized within blocks during training for each participant. Randomization was used to enhance overall motor learning during training (Maas et al., 2008). Two blocks of nonwords were created for each type of stimuli (similar, dissimilar) and generalization task (retention, transfer). These blocks were counterbalanced across participants. Figure 1 depicts a schematic example of the cross-over design of this study, the random assignment of nonwords (similar or dissimilar), and the counterbalancing used during the generalization tasks.

### Procedures

The experiment consisted of two nonword repetition tasks, each consisting of two parts: the “training” phase and the “generalization” phase. the experiment occurred in a quiet room where participants were seated in a chair at a table with a dynamic headset with a unidirectional microphone (SURE WH20XLR) placed approximately one inch from the participant’s mouth. The microphone connected to a digital voice recorder (Olympus DM-901), which recorded each nonword repetition task. All stimuli played through a stereo speaker (Bose Companion 2 Series 3) centered 15 in infront of the participant. The speaker was connected to a 64-bit Dell Latitude 3340 laptop with Windows 7 operating system, which ran Eprime (Schneider et al., 2002).

#### Training

Eprime randomly presented all training stimuli within a block. A total of 10 blocks of training were completed (i.e., 100 repetitions). All stimuli during training were of the same type (either similar or dissimilar). During a single trial, participants listened to an auditory presentation of a nonword and repeated the nonword into the microphone. The examiner, a graduate student in the WVU speech-language pathology program, perceptually rated the pronunciation of the participant’s nonword production and noted all incorrect productions by pushing a button on the laptop. At the end of 10 trials (or a single training block), summary feedback was provided by Eprime to enhance overall motor learning (Maas et al., 2008). Misarticulated nonwords were replayed through the speaker, and the participant was instructed to listen carefully to the repeated nonwords. Following summary feedback, training continued for another block. This procedure continued until all 10 blocks were completed.

#### Generalization

Following training, two generalization tasks using the same type of nonwords (similar or dissimilar) were administered: a retention task and a transfer task. For each task, 10 nonwords were presented *via* Eprime using the same nonword repetition procedure as used in training. No summary feedback was provided to participants following each task. The only difference between generalization tasks was the nonwords used. Trained stimuli were used in the retention task, and new nonwords (i.e., transfer nonwords) were used in the transfer task.

### Measurements

All nonword responses from training blocks 1 and 10, and all retention and transfer blocks, were individually scored for phoneme accuracy and coded as dichotomous variables (correct or incorrect; Shriberg and Kwiatkowski, 1982). Two blinded raters, trained in phonetic transcription, independently listened to the audio recordings of the participant’s nonword production and determined phoneme-by-phoneme accuracy by perceptual judgment. A third, blinded rater resolved any discrepancies in accuracy ratings. This point-by-point system forced 100% interscorer reliability.

Each phoneme was scored relative to the model production (total of seven phonemes per nonword). All phoneme distortions, substitutions, omissions, and insertions were considered incorrect. A percent phonemes correct (PPC) was calculated for each nonword by dividing the total number of correctly produced phonemes by the total number of phonemes. An average PPC score was calculated for each participant per nonword type (similar, dissimilar) and block (training blocks 1, 10, retention, transfer).

### Statistical Analyses

Two mixed-design analyses of variance (SPSS version 26) were used to evaluate the order of task administration (similar vs. dissimilar protocols) in relation to within-subject performance between different motor performance time points using similar and dissimilar nonwords. The first analysis was conducted to evaluate PPC score differences between different sets of stimuli during motor learning (blocks 1 and 10). The second analysis was conducted to evaluate PPC score differences between stimuli sets to determine if a contextual interference effect was observed (comparison of retention vs. transfer blocks). Outliers were observed with both analyses; however, all data were included in the analyses. Assumptions of normality were not met for all conditions in each analysis (*p* < 0.05). No correction was made as ANOVA statistics are typically robust to these violations. Assumptions of homogeneity of variance, homogeneity of covariances, and sphericity were met for both analyses. A significance level of 0.05 was used for hypothesis testing. Planned comparisons employed a Bonferroni correction, which adjusted the alpha level for multiple comparisons (0.05/4) for each analysis. All reported comparison significant values have already been adjusted using a Bonferroni correction and should be compared to a significance level of 0.05.

## Results

Data from 23 participants were included in the analyses. Subject attrition was due to failing one or more screening measures (*N* = 4) and an inability to finish the experimental protocol secondary to equipment failure (*N* = 3). Following attrition, 12 participants initiated training with similar nonwords, and 11 participants initiated training with dissimilar nonwords.

### Motor Learning

There was not a statistically significant interaction between the order of training (similar vs. dissimilar training) and PPC scores during blocks 1 and 10 regardless of stimuli type (similar or dissimilar nonwords), *F*(3,63) = 0.076, *p* = 0.972, partial *η* ^2^ = 0.004. No main effect was observed for order of training, *F*(1,21) < 0.001, *p* = 0.989, partial *η* ^2^ < 0.001. However, a main effect of training block showed that PPC scores were significantly different between blocks 1 and 10 when different stimuli were practiced, *F*(3,63) = 45.977, *p* < 0.001, partial *η* ^2^ = 0.686.

**Figure 2:**
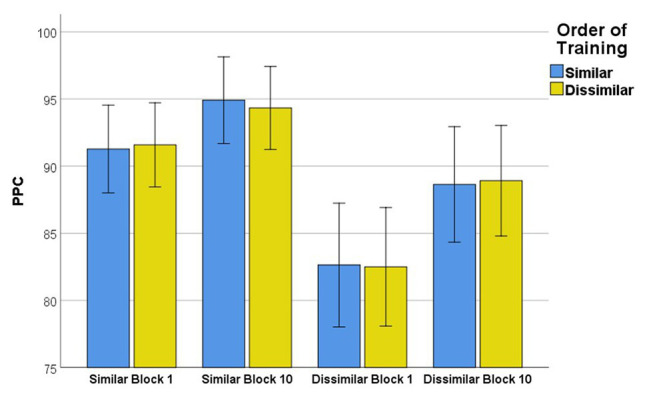
Mean phoneme accuracy for similar and dissimilar nonwords during training (blocks 1 and 10). Error bars - 95% CI.

Planned comparisons related to motor learning revealed PPC scores were significantly higher from blocks 1 to 10 when participants were practicing similar nonwords [*p* < 0.001, CI (−5.108, −1.279), *η* ^2^ = 0.497] and dissimilar nonwords [*p* < 0.001, CI (−9.015, −3.402), *η* ^2^ = 0.635]. At the end of the first block of training, participants were significantly more accurate in producing similar nonwords than dissimilar nonwords, *p* < 0.001, CI (5.699, 12.021), *η* ^2^ = 0.736. This difference in accuracy persisted through the end of training (block 10), where participants’ similar nonword productions were more accurately produced compared to dissimilar nonwords, *p* < 0.001, CI (2.979, 8.771), *η* ^2^ = 0.586. Figure 2 depicts average PPC scores and 95% confidence intervals for all stimuli.

### Contextual Interference Effect

There was not a statistically significant interaction between the order of training (similar vs. dissimilar training) and PPC scores during retention and transfer blocks regardless of stimuli type (similar or dissimilar nonwords), *F*(3,63) = 0.092, *p* = 0.964, partial *η* ^2^ = 0.004. No main effect was observed for order of training, *F*(1,21) = 0.786, *p* = 0.385, partial *η* ^2^ = 0.036. A main effect of generalization showed that PPC scores were significantly different between retention and transfer following practice with different stimuli, *F*(3,63) = 20.213, *p* < 0.001, partial *η* ^2^ = 0.49.

**Figure 3:**
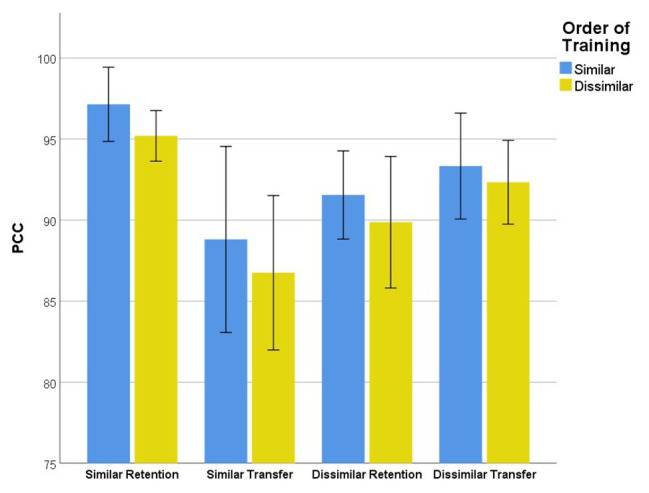
Mean phoneme accuracy for similar and dissimilar nonwords retention and transfer blocks. Error bars - 95% CI.

As noted in Figure 3, planned comparisons evaluating generalization of learning revealed a statistically significant decrease in PPC scores from the retention to transfer blocks when participants repeated similar nonwords [*p* < 0.001, CI (4.657, 12.118), *η* ^2^ = 0.642]. No significant difference in PPC scores was observed between retention and transfer blocks when participants repeated dissimilar nonwords [*p* = 0.056, CI (−4.995, 0.742), *η* ^2^ = 0.163].

Planned comparisons also revealed a contextual interference effect. Mean PPC scores were increased during the retention block when participants repeated similar nonwords compared to dissimilar nonwords, *p* < 0.001, CI (2.865, 8.054), *η* ^2^ = 0.611. Despite this gain, PPC scores were significantly lower when participants repeated new similar nonwords compared to new dissimilar nonwords during the transfer block, *p* < 0.001, CI (−8.363,-1.745), *η* ^2^ = 0.453.

## Discussion

This study investigated the contextual interference effect in speech motor learning by evaluating participants’ production accuracy during two nonword learning tasks. We had two predictions regarding participants’ speech motor learning. First, we predicted participants’ accuracy would increase with training (i.e., from blocks 1 to 10) regardless of stimuli type based on large amounts of random practice (Maas et al., 2008). This prediction was met. However, these effects were more significant when participants practiced similar nonwords compared to dissimilar nonwords. These results align with previous work where repeated, or identical phoneme sequences resulted in fewer speech production errors (Damian and Dumay, 2007; Mailend et al., 2019). Similarly, during speech perception training, practicing with fewer phoneme contrasts resulted in better acquisition during training (Nishi and Kewley-Port, 2007, 2008).

Secondly, we predicted that a contextual interference effect would be observed during the generalization task. The contextual interference effect is a phenomenon where performance is diminished during training when conditions create interference; however, this interference results in overall better generalization (Magill and Hall, 1990; Lee et al., 1992). The most well-researched variables of this effect include random vs. blocked practice (Shea and Morgan, 1979; Magill and Hall, 1990). We hypothesized for speech motor learning that similar and dissimilar phonemes might also produce this effect based on the mixed empirical results evaluating practice schedule during speech motor learning (Scheiner et al., 2014; Jones and Croot, 2016) and perceptual learning (Nishi and Kewley-Port, 2007, 2008). Specifically, we predicted that participants would have significantly higher phonemic accuracy repeating trained similar nonwords than dissimilar nonwords following training. However, this pattern would reverse when participants repeated new dissimilar transfer nonwords during the generalization task. This prediction was met. Participants were more accurate producing new dissimilar nonwords during the transfer task despite difficulties practicing with dissimilar nonwords during the training task.

Our results suggest the processing of repeated and similar phonemes within nonwords may impede speech motor learning. Practicing nonwords with similar phonemes may have resulted in the learner not discriminating unique features between stimuli during training. This lack of discrimination may have resulted in memory encoding of speech representations that lacked distinctive attributes. During the transfer block, retrieving memory representations from long-term memory resulted in decreased accuracy due to difficulty discriminating between similar memory representations (Lee, 1988). In contrast, participants practicing nonwords with different phonemes may have encoded distinctive memory representations. These distinctive features would allow for more efficient and accurate memory retrieval during the transfer stage of motor learning (Lin et al., 2018).

These results contrast to a language processing effect termed the “phoneme similarity effect,” where an increase in production errors was observed with repeated and similar phonemes (Yaniv et al., 1990; Rogers and Storkel, 1998; Wilshire, 1999). We did not anticipate the phoneme similarity effect to influence the motor learning outcomes in this study. However, the contrasting effect of repeated or similar phonemes on language vs. motor processing is interesting. During phonetic encoding, linguistic code transforms into a motor code executed by the speech system (Levelt et al., 1999). Nevertheless, the handoff between language and motor processing is not well understood (Laganaro, 2019). During this process, variables that inhibit language processing become facilitative during speech production. Further investigation is warranted to address the contexts and variables that may be altered by phonetic encoding. Moreover, there is also other evidence to suggest that language processing may enhance speech production.

Vowels are hyper articulated (i.e., an enlarged vowel space) when target words reside in dense lexical neighborhoods compared to sparse neighborhoods (Munson and Solomon, 2004; Wright, 2004; Watson and Munson, 2007). Similar vowel hyper articulation is observed if the target word comprises minimal pairs of phonemes, e.g., /b/ and /p/ (Peramunage et al., 2011). In these conditions, target words share similar phonemes. However, the phoneme similarity effect is not observed, and the overall articulation of the target word is enhanced. Fewer speech errors have also been reported when target words are from dense neighborhoods (Vitevitch, 2002; Vitevitch and Luce, 2004). Paradoxically, these effects reverse in broader contexts, such as conversation, where vowels became centralized and shortened in dense lexical neighborhoods (Gahl et al., 2012) or with frequently produced words (Fox et al., 2015). Thus, further research is essential in identifying the contexts and processing demands that shift linguistic influences from facilitating to inhibiting speech production.

There were limitations to our study. We used a carry-over design to evaluate the effect of phoneme similarity on speech motor learning. Our design limited the amount of time between each motor learning task, which may have created a potential carry-over effect between treatments. Although we attempted to minimize these effects with multiple sets of stimuli used for retention and transfer conditions, future studies should allow longer “wash out” periods between training sets. Moreover, this study investigated the immediate retention and transfer effects of training on two stimulus sets. This study provided a point of investigation for future studies evaluating lasting motor changes, where at least a day between training and generalizations tasks would be observed (Kantak and Winstein, 2012).

The use of pre-constructed stimuli, used in prior speech motor learning paradigms, ensured that participants with intact speech abilities would learn a novel speech-like task during this experiment. However, this limited the amount of control over how similar phonemes were based on multiple indexes of similarity. The findings from this study provide a preliminary definition of “similarity” that may be manipulated in future studies, further exploring the contextual interference effect in speech motor control. Other factors may include changes in construction related to consonant age of acquisition, manner and place of articulation, or voicing features of phonemes (Bailey and Hahn, 2005). Further evaluation of similarity indices in constructing speech stimuli is needed to understand better how phonemic properties may influence speech motor learning.

## Data Availability Statement

The raw data supporting the conclusions of this article will be made available by the authors, without undue reservation.

## Ethics Statement

The studies involving human participants were reviewed and approved by Institutional Review Board at West Virginia University. The participants provided their written informed consent to participate in this study.

## Author Contributions

Both the authors listed have made a substantial, direct, and intellectual contribution to the work and approved it for publication.

### Conflict of Interest

The authors declare that the research was conducted in the absence of any commercial or financial relationships that could be construed as a potential conflict of interest.
